# Acute Human Inkoo and Chatanga Virus Infections, Finland

**DOI:** 10.3201/eid2205.151015

**Published:** 2016-05

**Authors:** Niina Putkuri, Anu Kantele, Lev Levanov, Ilkka Kivistö, Markus Brummer-Korvenkontio, Antti Vaheri, Olli Vapalahti

**Affiliations:** University of Helsinki, Helsinki, Finland (N. Putkuri, L. Levanov, I. Kivistö, M. Brummer-Korvenkontio, A. Vaheri, O. Vapalahti);; Helsinki University Hospital, Helsinki (A. Kantele, A. Vaheri, O. Vapalahti)

**Keywords:** California serogroup, orthobunyavirus, Inkoo virus, Chatanga virus, California encephalitis virus group, clinical infection, Bunyaviridae infection, arbovirus encephalitis, viruses, Finland, vector-borne infections

## Abstract

Inkoo virus (INKV) and Chatanga virus (CHATV), which are circulating in Finland, are mosquitoborne California serogroup orthobunyaviruses that have a high seroprevalence among humans. Worldwide, INKV infection has been poorly described, and CHATV infection has been unknown. Using serum samples collected in Finland from 7,961 patients suspected of having viral neurologic disease or Puumala virus infection during the summers of 2001–2013, we analyzed the samples to detect California serogroup infections. IgM seropositivity revealed 17 acute infections, and cross-neutralization tests confirmed presence of INKV or CHATV infections. All children (<16 years of age) with INKV infection were hospitalized; adults were outpatients with mild disease, except for 1 who was hospitalized with CHATV infection. Symptoms included fever, influenza-like illness, nausea or vomiting, disorientation, nuchal rigidity, headache, drowsiness, and seizures. Although many INKV and CHATV infections appear to be subclinical, these viruses can cause more severe disease, especially in children.

## Abstract 2

Most cases appeared to be subclinical, but a few patients, usually children, required hospitalization.

Inkoo virus (INKV) and Chatanga virus (CHATV) are 2 members of the California serogroup of orthobunyaviruses that are currently found in Finland. They are trisegmented, enveloped negative-strand RNA viruses belonging to genus *Orthobunyavirus* (family *Bunyaviridae*), which includes several recognized mosquitoborne human pathogens. 

INKV was first isolated from *Ochlerotatus communis* and *O.* *punctor* mosquitoes in 1964 in Finland (*1*) and has since been found in Sweden, Norway, and Russia (*2**–**4*). The high seroprevalence in these countries suggests that INKV infections are common in these locations (*5**–**8*). Although the virus has been known to occur in Finland for decades, only 1 domestic report describes a possible association of INKV to clinical disease (*9*). Reports from Russia show INKV IgM or neutralizing antibodies in patients with neurologic symptoms or fever, but only 2 cases were identified as INKV infection; most often, the California serogroup virus infections were caused by Tahyna virus (TAHV), or the infecting virus could not be defined (*10**–**12*). 

CHATV was isolated from mosquitoes collected in Finland in 2007 (*13*) but is known to have circulated earlier in Russia, where the first characterized isolate was from a mosquito collected in 1987 (*14*). CHATV strains have ≈84% aa identity with INKV within the nucleocapsid protein but are more similar to the Snowshoe hare virus (93% nucleocapsid protein identity) that occurs in the United States. We found no previous reports of CHATV infections naturally occurring in humans or animals.

Patients with California serogroup virus infections usually remain asymptomatic or have symptoms of mild influenza-like illness, but some of these viruses may also cause encephalitis (*15**–**17*). The California serogroup viruses cross-react on many serologic tests, so neutralization assays are required to verify the specific virus. In the United States, 29–167 cases of California serogroup virus encephalitis are diagnosed annually, and most cases result from La Crosse virus (LACV) (*18*), which is one of the most important arboviral agents causing encephalitis in children in the United States but is rarely found in adults. This pattern contrasts with the arbovirus West Nile virus, which causes central nervous system (CNS) infections in adults more often than in children (*19*). LACV encephalitis can be mistaken for herpes simplex virus (HSV) or enterovirus meningoencephalitis and is often undiagnosed (*15**,**20*). Other California serogroup viruses that cause neuroinvasive disease in the United States and Canada are California encephalitis, Jamestown Canyon, and Snowshoe hare viruses (*17**,**21**–**24*), although infections caused by these viruses are reported more rarely than those caused by LACV and are not as extensively studied.

The incidence of California serogroup virus infections in Europe is largely unknown because of underdiagnosing and underreporting that result from lack of alertness among healthcare workers and lack of surveillance efforts. Available data indicate that TAHV has the most widespread distribution in Europe and is mostly asymptomatic or causes febrile illness, especially in children (*16**,**25**,**26*).

Because pathogens that cause encephalitis during the summer months in Finland are mostly unknown (*27*), we attempted to study the occurrence of acute California serogroup virus infections, particularly those caused by INKV, in febrile and encephalitic patients during the mosquito season in Finland and to characterize those infections. We report our observance of INKV and CHATV infections in humans and describe the clinical characteristics of acute infections caused by these viruses.

## Methods and Materials 

### Patient Samples

Our analysis comprised 3 sets of patient samples (7,961 total patients). First, we retrospectively screened serum samples that were collected from patients in healthcare facilities across Finland during the summer months of 2001–2013 (Table 1) and were sent as diagnostic samples to the Department of Virology and Immunology, Helsinki University Central Hospital Laboratory, Hospital District of Helsinki and Uusimaa (institutional review board permit 119/E0/05). For patients presumed to have CNS symptoms, samples were screened for antibodies against a panel of meningoencephalitis agents (HSV, varicella zoster virus, human herpesvirus 6, enterovirus, and *Mycoplasma pneumoniae* bacteria); for patients presumed to have febrile illness, samples were screened for Puumala virus. For most samples, laboratory screening was negative for the viral agents studied. In addition to these 2 sample groups, we analyzed samples specifically received for screening of INKV antibodies during the study period. 

**Table 1:** Serum samples screened for California serogroup virus IgM and IgM-positive samples, by patient group and date of collection, Finland, 2001–2013*

Patient group and date of sample collection†	Serum samples, no.	IgM-positive samples, no.	IgM prevalence, %
Suspected Puumala virus infection			
2001 May 25−Sep 4	1,294	2	0.15
2004 Jun 14–Sep 1	958	1	0.10
2012 Jun 5–Aug 21	498	2	0.40
2013 May 16–Sep 26	824	0	0
Total	3,574	5	0.14
Neurologic symptoms			
2003 Jun 2–Sep 29	711	2	0.28
2004 Jun 10–Sep 17	868	2	0.23
2005 Jun 23–Oct 1	969	2	0.21
2007 Jun 20–Aug 30	563	0	0
2012 Jun 8–Oct 15	1,103	3	0.27
Total	4,214	9	0.21
Suspected Inkoo virus infection			
2004	32	1	3.13
2005	30	0	0
2006	21	0	0
2007	31	0	0
2008	11	0	0
2009	15	0	0
2010	16	0	0
2011	14	0	0
2012	3	0	0
Total	173	1	0.58
All patient groups	7,961	15	0.19
*Samples from patients were initially screened for Puumala virus, for agents causing neurologic infections, or Inkoo virus. Indirect immunofluorescence was used to screen for California serogroup virus IgM. †Sample collection for suspected Inkoo virus infection was for the entire year.			

Of the total 8,793 samples we tested for California serogroup virus IgM, 4,214 serum samples and 832 cerebrospinal fluid (CSF) samples had been initially sent for screening of meningoencephalitis agents; 3,574 serum samples had been initially sent for Puumala virus testing; and 173 serum samples had been sent for INKV testing. Samples were stored at −20°C; aliquots of the serum samples were stored at –70°C for PCR testing. Laboratory data and patient histories were collected from patient records for cases with confirmed California serogroup virus IgM positivity. Data on 2 previously confirmed cases with INKV infection that occurred in 1976 and 1980 were included in the analysis; these cases had been confirmed with hemagglutination inhibition and neutralization tests, and full patient histories had been described previously (*9*).

### Serologic Testing

Serum samples were screened with an indirect INKV-IgM immunofluorescence (IFA) test described previously (*5*). In brief, IFA slides contained Vero E6 cells (green monkey kidney cells, American Type Culture Collection, CRL-1586, Manassas, VA, USA); 30% of the cells were infected with INKV. Samples (serum diluted at 1:20 ratio; CSF undiluted) were incubated on slides overnight and then washed with phosphate-buffered saline; anti–human IgM fluorescein isothiocyanate conjugate was then added, and samples were incubated for 1 hour. After being washed with phosphate-buffered saline, slides were dried and examined with a fluorescence microscope. IgM-positive samples were retested with IgM IFA after removal of IgG by using Gullsorb treatment (Meridian Bioscience, Inc., Cincinnati, OH, USA) for the serum samples. To ensure that diagnostic criteria of acute infection were met, as previously described, IgM-positive samples were confirmed with IgG IFA testing (*5*) and studied with INKV prototype strain KN3641 (*1*), CHATV Möhkö strain M07–1 (*13*), and TAHV prototype strain Bardos 92 (*28*) plaque-reduction neutralization test (PRNT) (*13*). PRNT was performed because of the occurrence of cross-reactions. 

### Reverse Transcription PCR

When possible (i.e., when sufficient sample remained after serologic testing), IgM-positive samples were tested with reverse transcription PCR (RT-PCR) to study the presence and kinetics of viremia and to obtain information on the viral sequences. RNA extraction was performed by using the QIAamp Viral RNA Mini Kit (QIAGEN, Valencia, CA, USA) according to the manufacturer’s instructions. The extracted RNA was reverse transcribed to cDNA with ERT-Ro Roche Expand Reverse Transcriptase (Roche, Indianapolis, IN, USA). PCR methods, as described previously (*29**,**30*), were modified to work with the Phusion Flash High-Fidelity PCR Master Mix (Thermo Scientific, Grand Island, NY, USA). The mixture included 1.25 µL of each primer (10 µmol/L), 12.5 µL of the Phusion Flash 2X buffer, and 8 µL of sterile distilled deionized water, producing a total volume of 25 µL, which included 2 µL of cDNA.

## Results

Our hospital diagnostic laboratory received 7,961 serum and 832 CSF samples from 7,961 patients in Finland during 2001–2013 (Table 1). Samples were initially submitted to determine antibodies against either a panel of agents causing neurologic infections (4,214 serum and 832 CSF samples), Puumala virus (3,574 serum samples), or INKV (173 serum samples) (Table 1). Most (4,299 [54%]) serum samples were from healthcare settings in southern Finland. Children <10 years of age had the smallest number of samples; adults 50–59 years of age had the largest number of samples. A slight preponderance (52%) of sampled patients were male. 

Including the 2 previously confirmed cases, a total of 17 serum samples and no CSF samples were found IgM positive for California serogroup viruses. Serum samples were IgM positive among 0.21% of patients with CNS symptoms, 0.14% of patients with suspected Puumala virus infection, and 0.58% of patients with suspected INKV infection (Table 1). Frequency of IgM positivity was similar for different years of sample collection. The age range of patients with positive results was 7–81 years; 8 patients were female and 9 were male. California serogroup viruses IgM was found most commonly in patients <19 or 50–59 years of age (Table 2). Most (88%) IgM-positive cases were detected during or after late August. Fourteen (82%) of the 17 patients showed the highest neutralizing antibody titers (up to 1,280) against INKV, whereas 3 patients showed the highest titers (up to 20,480) against CHATV. Neutralization tests for 2 of the 3 CHATV patients resulted in titers >4 times those for other studied California serogroup viruses, which met the diagnostic criterion for confirmed CHATV infection; the third CHATV patient with a lower titer likely had CHATV infection (Table 3). The earliest that IgM was detectable was day 3 of symptom onset (fever); 1 patient still had detectable IgM 3 weeks after symptom onset (Table 4). IgM titers varied generally between 1:30 and 1:160, and exceeded 1:320 in only 3 patients. IgG in IFA was detected in most IgM-positive patients. No patient had detectable California serogroup virus RNA.

**Table 2:** Age group and sex of patients whose serum samples were tested and number of samples that were IgM positive for INKV and CHATV infections in Finland, 2001–2013*

Characteristic	Patients, no. (%)	INKV infection	CHATV infection
Age range			
0–9	670 (8.42)	1	0
10–19	717 (9.01)	2	0
20–29	905 (11.37)	0	0
30–39	1062 (13.34)	1	0
40–49	1180 (14.82)	2	1
50–59	1377 (17.30)	5	1
60–69	1093 (13.73)	1	0
>70	957 (12.02)	0	1
Sex			
F	3802 (47. 76)	8	0
M	4159 (52.24)	6	3
*CHATV, Chatanga virus; INKV, Inkoo virus.			

**Table 3:** Clinical concurrent conditions and immunofluorescence and neutralization titers of patients with IgM-positive California serogroup virus infections in Finland, 2001–2013*

Patient no.	IgG IFA titer	IgM IFA titer	PCR result	Underlying illness	Reason for medical care	PRNT titer		
						INKV	CHATV	TAHV
1	160	160	Neg	–	Hospitalized (unknown infection)†	320	40	40
2	80	40	Neg	–	Hospitalized (unknown infection)†	320	640‡	40
3	120	40	Neg	–	Hospitalized (unknown infection)†	320	40	40
4	>640	>320	Neg	Hypertension	Hospitalized (unknown infection)†	320	20,480	5,120
5§	160	+/ND¶	ND	–	Hospitalized (unknown infection)†	ND	ND	ND
6§	320	+/ND¶	ND	–	Hospitalized (unknown infection)†	ND	ND	ND
7	80	>320	Neg	Type 2 diabetes, hypertension	Fever (unknown infection)†	>640	40	80
8	320	40	Neg	–	No information	320	<20	<20
9	80	80	Neg	Asthma, immunodeficiency	No information	160	40	<40
10	40	20	ND	Hypothyroidism	Follow-up visit (suspected MS, neurologic disorder)	160	20	40
11	320	>320	Neg	–	Follow-up visit (recurrent respiratory tract infections for 4 mo, suspected immunodeficiency)	320	40	80
12	<20	40	Neg	Schizophrenia, hypothyroidism	Follow-up visit (HSV eye infection, rash, Steven-Johnson syndrome)	320	20	40
13	160	120	Neg	–	Hospitalized, acute infection (*E. coli* urosepsis)	160	40	40
14	<20	120	Neg	MS disease, hypothyroidism	Hospitalized, acute infection (HSV infection)	>640	<20	20
15	320	120	Neg	–	Hospitalized, acute infection (impetigo contagiosa)	640	40	40
16	960	40	ND	–	Hospitalized, multiple infarcts in the central nervous system	1,280	5,120	1,280
17	80	30	Neg	–	Hospitalized, epidemic nephropathy	320	<20	<20

*CHATV, Chatanga virus; HSV, herpes viruses; IFA, indirect immunofluorescence; INKV, Inkoo virus; MS, multiple sclerosis; ND, PCR not done; PRNT, plaque reduction neutralization test; TAHV, Tahyna virus; –, no underlying illness.  †Full patient history describing INKV or CHATV infection. ‡4-fold difference between titers was not achieved with neutralization test, the diagnostic criterion used to confirm CHATV infection. §INKV infection confirmed by hemagglutination inhibition test and neutralization test earlier in Helsinki University Central Hospital laboratory. ¶Samples tested were IgM positive, but titer was not tested.

**Table 4:** Clinical progression of illness for patients hospitalized with acute INKV (n = 4) or CHATV infection (n = 2), Finland*

Virus and patient no.	Illness progression	Additional findings
INKV		
1	Day 1: fever 38°C, influenza-like symptoms	Elevated HHV-6 antibody levels from same sample
	Day 3: disoriented	
	Day 6: hospitalized, abnormal EEG, CAL IgM+	
	Day 7: psychotic but discharged	
	Day 10: follow-up EEG shows same abnormalities	
	3 mo later: EEG almost normal	
2	Day 1: fever 39.5°C, headache, nuchal rigidity, sore throat before fever, hospitalized	Tick bite 1 mo earlier, erythema migrans; day 1: BorrAb neg
	Day 2: nuchal rigidity, headache deteriorating, slowness but oriented	
	Day 3: discharged, CAL IgM+	
	Day 5: headache again, hospitalized	
	Day 6: discharged	
3	Day 1: vomiting	
	Day 2: stomach pain, diarrhea, seizures, hospitalized	
	Day 3: fever 38.3°C, drowsiness, convulsions	
	Day 4: More seizures, small changes in EEG	
	Day 5: CAL IgM+	
	Day 8: discharged	
4	Day 1: fever 37.9°C, sore throat	Tick bite 3 wks earlier
	Day 3: CAL IgM+	
	Day 4: nausea and vomiting	
	Day 5: fever 39°C, headache, nuchal rigidity, hospitalized	
	Day 10: recovered and discharged	
CHATV		
1	Day 1: vomiting continuing for 3 d	
	Day 4: fever, hospitalized, disoriented at night	
	Day 7: frontal headache, normal head CT and abdominal ultrasound	
	Day 12: discharged, CAL IgM+	
2	Day 1: fever 39°C, back pain	Back injury 2 wks earlier
	Day 7: hospitalized, high fever, back pain almost resolved	
	Day 7–22: temporal pain, trembling of hands, fluctuating fever	
	Day 17: CAL IgM+	
	Day 23: discharged	

*BorrAb, Borrelia antibody test; CHATV, Chatanga virus; CT, computer tomographic scan; EEG, electroencephalogram; HHV, human herpesvirus; INKV, Inkoo virus. CAL IgM+ indicates the day when IgM for California encephalitis group viruses was observed.

Clinical histories were collected for all patients whose samples had a positive California serogroup virus IgM result (Table 3). Patients could be divided into 2 groups: those with a known reason (other than INKV or CHATV infection) for seeking medical care (10/17) and those for whom the cause of acute infection was unknown (7/17) (Table 3). The latter group of patients were evaluated for California serogroup virus infection because their medical records showed no other cause for their symptoms (i.e., no underlying disease or laboratory findings that implied another infection) (Table 4). All 4 children (<16 years of age) with an unknown infection had acute INKV infection, whereas 2 adults with an unidentified infection had CHATV infection. These 6 patients were hospitalized. All had fever and other symptoms such as sore throat, nausea and vomiting, and neurologic conditions such as disorientation, nuchal rigidity, headache, and drowsiness. Small changes in electroencephalography were observed in 2 patients, and 1 patient had seizures (Tables 4, 5). All patients fully recovered from the infections.

**Table 5:** Symptoms of acute INKV and CHATV infections, as recorded in charts of 7 patients hospitalized with unknown infection, Finland*

Symptom	INKV, N = 5	CHATV, N = 2
Fever	5	2
Influenza-like symptom	4	0
Headache	4	2
Nausea/vomiting	2	1
Disorientation	2	1
Sore throat	2	0
Nuchal rigidity	2	0
Changes in EEG	2	0
Diarrhea	1	0
Seizure	1	0
Drowsiness	1	0

*These 7 patients were hospitalized with unknown infection (Table 3), which were determined to be Inkoo virus (INKV) or Chatanga (CHATV) infections. Symptoms are listed in order of frequency. EEG, electroencephalogram.

Of the 17 California serogoup virus infection IgM-positive patients, 11 were not hospitalized for that infection. Four of these 11 patients visited a physician only once; 6 others were treated for another indication because their symptoms were interpreted as resulting from causes other than INKV or CHATV infection. Ten (91%) of the 11 patients who were not hospitalized were >40 years of age. Six (55%) of the 11 outpatients had a laboratory-confirmed co-infection with another pathogen, such as HSV (antigen positive), acute Puumala virus infection (i.e., the same serum sample was positive for Puumala virus IgM), an *Escherichia coli* urosepsis, and impetigo contagiosa. One patient was reported to have had erythema migrans 4 weeks before sample collection, yet no antibodies against *Borrelia burgdorferi* were found. One patient suffered a back injury 1 week before symptom onset (Table 3).

## Discussion

In previous studies, the prevalence of California serogroup virus antibodies was high (30%–40%) in Nordic countries (*5**,**8*). To maintain such a high seroprevalence in Finland, >20,000 acute infections would need to occur annually during the mosquito season. Worldwide, California serogroup viruses other than INKV and CHATV have been associated with febrile illnesses and neurologic infection, but these infections have not been characterized in Finland. For that purpose, we retrospectively screened panels of serum samples that were originally collected over a period of years during the mosquito season and sent to our diagnostic laboratory for detection of antibodies to either causative agents of CNS infection (HSV1, HSV2, human herpesvirus 6, varicella zoster virus, *Mycoplasma pneumoniae*), Puumala virus, or INKV.

We estimated the frequency of acute human CHATV and INKV infections and characterized symptoms of these infections. The 2 patients with confirmed CHATV infection show that CHATV can cause human infection. We found symptoms that were similar to those reported for other viruses in the California serogroup. Both INKV and CHATV have remained practically unknown among physicians in Finland, and the rate of clinical suspicion has been negligible here (Table 1). The lack of awareness regarding these infections is similar to the situation with California serogroup viruses in many other countries.

A few articles from Russia have described the outcome and neurologic characteristics of INKV infection (*10**–**12*). In these studies, most patients had fever; almost 30% had neurologic symptoms (*11*). Studies in the Ryazan area showed that INKV infection was most frequently found in adults 21–40 years of age, and the number of cases peaked during the 2 periods of May and early August (*31*). In our study, 88% of the INKV cases were found during August and September or even later; only 2 cases occurred in early summer, and most patients with acute infection were <16 or 50–59 years of age. Another study from Russia included 520 selected patients; overall, 9.8% had California serogroup infection, with 2.5% and 1.2% INKV incidence in febrile and encephalitis patients, respectively (*11*). A more detailed study on the symptoms of the California serogroup infection included 118 patients, but INKV was confirmed in only 2 patients, 1 with multiple sclerosis and the other with meningoencephalitis (*10*). In that study, TAHV and undefined California serogroup infections (i.e., neutralization tests found no difference between INKV and TAHV) occurred more frequently than INKV (*10*). However, during 1995, the study period, CHATV had not yet been isolated but, as we now know, was already circulating in the area (*14*). Consequently, these infections could have been caused by CHATV. 

Our study confirmed both INKV and CHATV infections in Finland. Most identified acute cases were from the Helsinki hospital district (Figure), possibly because most samples were collected in southern Finland, where the laboratory is located and where Finland’s population density is highest. The overrepresentation of samples from the southern region may have decreased the likelihood of finding acute cases because distribution of California serogroup virus seroprevalence among humans is greater in northern Finland than in southern Finland, although the seroprevalence has recently increased in southern parts of the country (*5**,**32*). Furthermore, the high seroprevalence suggests that the serogroup viruses are found abundantly in nature and that infection is fairly common, so the frequency of acute cases we observed may underestimate the actual number of cases in Finland. Most acute cases occurred in autumn, yet the population density of the INKV principal vector, *O. communis*, is highest in June. Further studies are needed to determine whether this time lag reflects a long incubation period, spillover to other vectors, replication cycle in amplification hosts, or a change in vector occurrence.

**Figure:**
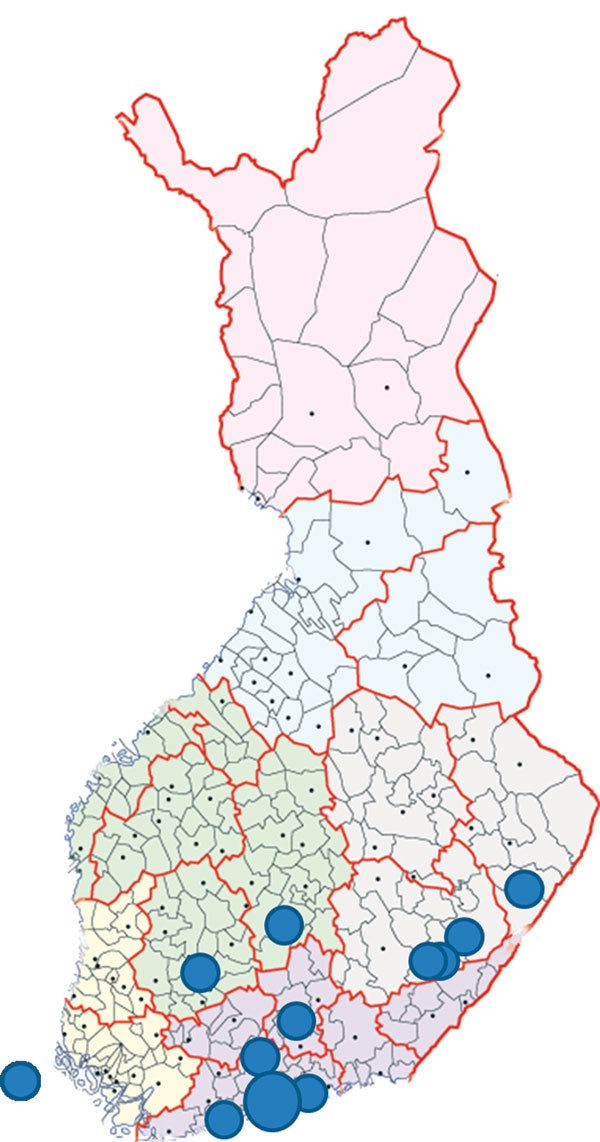
Locations of residence for 17 patients who were IgM positive for California serogroup virus infections, Finland. Each dot represents 1 patient except for the largest dot in southern Finland, which indicates a site for 6 patients. The dot on the far left indicates a patient from Åland Islands, Finland. Map source: National Land Survey of Finland (© 2015).

The high seroprevalence in Finland with the low frequency of cases requiring healthcare and low frequency of diagnostic sampling suggests that most of these infections are subclinical or manifest as mild disease. On the other hand, the patient panels used in our study were highly selected, so we could have missed INKV and CHATV cases among patients with other symptom patterns. In addition, the IgM IFA test used may not have been sensitive enough to detect IgM in all cases. The high seroprevalence in the populations in Finland may also be influenced by the newly identified California serogroup virus isolate found here, the Chatanga virus Möhkö strain (*13*). Results from the diagnostic test in use show cross-reactions between the serogroup viruses. Most (82%) of the acute infections in this study were INKV cases, confirmed by using PRNT, which showed >4-fold titer differences, consistent with findings that INKV is the major California serogroup agent in seroprevalence studies in Finland and Sweden (*33*). Although most cases seem subclinical or mild, the data in our study indicate that the clinical disease may occasionally be severe; all children with acute illness had INKV infection and were hospitalized. Although INKV infection in adults was mild, 1 adult patient with CHATV infection required hospital care.

The considerable variation in IgG and IgM titers of serum samples from patients with acute infection may suggest that the samples were taken at different stages of the acute infection, but the differences may simply reflect variation in the antibody levels in individual patients. IgM was detectable in several patients concomitantly with high titers of IgG and neutralizing antibodies, a finding suggesting that IgM may persist for several weeks. One patient had detectable IgM 17 days after symptom onset (Table 4). A follow-up sample would be needed to show seroconversion and confirm the acute infection. No patient samples were collected during the viremic stage, and all samples were RT-PCR negative. Distinct LACV strains are known to cause different symptoms (*34*), but neither INKV nor CHATV have been isolated from a human sample. Human isolates or sequences of these viruses would be valuable for comparing the pathogenicity of the strains and analyzing cases in patients requiring hospitalization. 

Many of our cases appear to represent recent subclinical infections that were identified only because of IgM testing and were unrelated to the reason for patients’ visits to the healthcare unit. In the 2 patients with HSV cold sores, subclinical INKV infection could have triggered reactivation of HSV. Alternatively, INKV may require the presence of an underlying disease or trauma to cause a symptomatic infection (e.g., by enabling the virus to cross the blood–brain barrier). Some reports from Russia suggest that certain concomitant microbial infections may render the course of the INKV infection more severe (*11*).

In conclusion, we describe INKV and CHATV infections in humans and the clinical characteristics of acute disease. Symptoms of acute INKV and CHATV infections in patients in our study resembled symptoms of other California serogroup virus infections: influenza-like illness, with fever being most prominent. Most acute cases appeared to be subclinical, and a small minority of patients required hospitalization. Compared with adults, children were at higher risk for contracting more severe disease and were more often hospitalized because of INKV infection. In adults, CHATV infection appeared to be more severe than INKV infection. Further studies are required to explore in detail the clinical picture, prognosis, incubation period, and antibody kinetics of these infections. Viral isolates or RT-PCR–positive samples from patients are needed to acquire data related to INKV and CHATV strains causing the clinical cases.
